# Supplementary material for: From sequence to enzyme mechanism using multi-label machine learning
Source: BMC Bioinformatics. 2014 May 19;15:150. doi: 10.1186/1471-2105-15-150 (PMC4229970; doi:10.1186/1471-2105-15-150)
Supplement: Additional file 2 — Java code of ml2db. Additional file ml2db_code.tar.gz contains the Java source code to run the multi-label machine learning experiments and save the results to database. The code’s Javadoc is included. [file 1471-2105-15-150-S2.zip › additional file 2/ml2db/ml2db/doc/mulan/classifier/meta/HMCNode.html]

HMCNode


JavaScript is disabled on your browser.


- Overview
- Package
- Class
- Use
- Tree
- Deprecated
- Index
- Help

- Prev Class
- Next Class

- Frames
- No Frames

- All Classes

- Summary:
- Nested |
- Field |
- Constr |
- Method

- Detail:
- Field |
- Constr |
- Method


mulan.classifier.meta

## Class HMCNode

- java.lang.Object
- - mulan.classifier.MultiLabelLearnerBase
  - - mulan.classifier.meta.MultiLabelMetaLearner
    - - mulan.classifier.meta.HMCNode

- All Implemented Interfaces:
  :   java.io.Serializable, MultiLabelLearner, weka.core.TechnicalInformationHandler

  ---

    

  ```
  public class HMCNode
  extends MultiLabelMetaLearner
  ```

  Class that implements a node for the `HMC`

  Version:
  :   0.2

  Author:
  :   George Saridis

  See Also:
  :   Serialized Form

- - ### Constructor Summary

    Constructors

    Constructor and Description

    `HMCNode(java.lang.String name,
    MultiLabelLearner mlc)`

    Creates a new instance with the given name and learner
  - ### Method Summary

    Methods

    | Modifier and Type | Method and Description |
    | --- | --- |
    | `void` | `addChild(HMCNode child)` Adds a child to the node. |
    | `java.util.HashSet<HMCNode>` | `getChildren()` Returns the children of the current node |
    | `java.util.ArrayList<java.lang.String>` | `getChildrenLabels()` Returns a list of all children labels |
    | `java.util.ArrayList<java.lang.String>` | `getDescendantLabels()` Returns all descendant labels |
    | `weka.core.Instances` | `getHeader()` Returns the header information |
    | `int[]` | `getLabelIndices()` Returns the label indices |
    | `java.lang.String` | `getName()` Returns the name of a node |
    | `int` | `getNumChildren()` Returns the number of children |
    | `weka.core.TechnicalInformation` | `getTechnicalInformation()` Returns an instance of a TechnicalInformation object, containing detailed information about the technical background of this class, e.g., paper reference or book this class is based on. |
    | `java.lang.String` | `globalInfo()` Returns a string describing the multi-label learner. |
    | `boolean` | `hasChildren()` Checks whether the node has children |

    - ### Methods inherited from class mulan.classifier.meta.MultiLabelMetaLearner

      `getBaseLearner`
    - ### Methods inherited from class mulan.classifier.MultiLabelLearnerBase

      `build, getDebug, isUpdatable, makeCopy, makePrediction, setDebug`
    - ### Methods inherited from class java.lang.Object

      `equals, getClass, hashCode, notify, notifyAll, toString, wait, wait, wait`

- - ### Constructor Detail


    - #### HMCNode

      ```
      public HMCNode(java.lang.String name,
             MultiLabelLearner mlc)
              throws java.lang.Exception
      ```

      Creates a new instance with the given name and learner

      Parameters:
      :   `name` - name of the node
      :   `mlc` - learner

      Throws:
      :   `java.lang.Exception`
  - ### Method Detail


    - #### getLabelIndices

      ```
      public int[] getLabelIndices()
      ```

      Returns the label indices

      Returns:
      :   label indices


    - #### getHeader

      ```
      public weka.core.Instances getHeader()
      ```

      Returns the header information

      Returns:
      :   header


    - #### hasChildren

      ```
      public boolean hasChildren()
      ```

      Checks whether the node has children

      Returns:
      :   whether the node has children


    - #### getNumChildren

      ```
      public int getNumChildren()
      ```

      Returns the number of children

      Returns:
      :   number of children


    - #### getChildren

      ```
      public java.util.HashSet<HMCNode> getChildren()
      ```

      Returns the children of the current node

      Returns:
      :   a set of nodes


    - #### getChildrenLabels

      ```
      public java.util.ArrayList<java.lang.String> getChildrenLabels()
      ```

      Returns a list of all children labels

      Returns:
      :   list of children labels


    - #### getDescendantLabels

      ```
      public java.util.ArrayList<java.lang.String> getDescendantLabels()
      ```

      Returns all descendant labels

      Returns:
      :   a list of descendant labels


    - #### addChild

      ```
      public void addChild(HMCNode child)
      ```

      Adds a child to the node.

      Parameters:
      :   `child` - - the child that will be added


    - #### getName

      ```
      public java.lang.String getName()
      ```

      Returns the name of a node

      Returns:
      :   name of the node


    - #### getTechnicalInformation

      ```
      public weka.core.TechnicalInformation getTechnicalInformation()
      ```

      **Description copied from class: `MultiLabelLearnerBase`**

      Returns an instance of a TechnicalInformation object, containing detailed
      information about the technical background of this class, e.g., paper
      reference or book this class is based on.

      **Specified by:**
      :   `getTechnicalInformation` in interface `weka.core.TechnicalInformationHandler`

      **Specified by:**
      :   `getTechnicalInformation` in class `MultiLabelLearnerBase`

      Returns:
      :   the technical information about this class


    - #### globalInfo

      ```
      public java.lang.String globalInfo()
      ```

      **Description copied from class: `MultiLabelLearnerBase`**

      Returns a string describing the multi-label learner.

      **Specified by:**
      :   `globalInfo` in class `MultiLabelLearnerBase`

      Returns:
      :   a description suitable for displaying in a future gui


- Overview
- Package
- Class
- Use
- Tree
- Deprecated
- Index
- Help

- Prev Class
- Next Class

- Frames
- No Frames

- All Classes

- Summary:
- Nested |
- Field |
- Constr |
- Method

- Detail:
- Field |
- Constr |
- Method
